# Green Vardman Black, D.D.S., M.D., S.C.D., L. L. D.

**Published:** 1915-10

**Authors:** 


### OBITUARY.

GREEN VARDMAN BLACK, D.D.S., M.D., S.C.D., L. L. D.—Dr. Black was born in Winchester, Ills., Aug. 3, 1836. He died in "Walnut Lodge," his paternal home, near Jacksonville, Ills., Tuesday, Aug. 31, 1915. His death was caused by pernicious anemia, from which he had suffered for some time. His feeble health and probably a conscious feeling that he would not live much longer impelled him to leave his home in Chicago and go to the old farm which he owned and which had meant so much to him all his life, as it was his recreation or play ground. He lived only two weeks after reaching his old home. Dr. Black's parents came originally from North Carolina. He left home when he was about sixteen years old and went to live with his brother, Dr. T. G. Black, a physician of Clayton, Ill., with the intention of reading medicine. When he was twenty-one years old he changed his plans and took up the study of dentistry with Dr. J. C. Speer of Mt. Sterling, Ills., and began the practice of dentistry in Winchester, Ills. He married Jane L. Coughennower in 1860, who died in 1863. In 1865 he married Elizabeth Akers Davenport, who survives him with the following children, Dr. Carl E. Black, Miss Clara Black, Dr. Arthur D. Black, and Mrs. Mark Baldwin. Dr. Black served as a sergeant in the Federal army in 1862 and 1863, and because of a knee joint injury he was discharged the latter part of 1863 and located in Jacksonville, Ills., for the practice of dentistry in 1864, where he remained until 1897, when he removed to Chicago, and became Dean of the American Dental College—now the Dental School of Northwestern University—which position he retained until the time of his death. He has been one of the most conspicuous and versatile members of the dental profession in all its history. His works have been both technical and scientific, and no writer on dentistry has been so prolific. We could not catalogue the tremendous work done by this great man, in the space at our command. This was done a few years ago in a pamphlet prepared by his son, Dr. A. D. Black, for the great testimonial banquet given him in Chicago, January 29, 1910, since which time his great book on Dental Pathology has been published, which is the most comprehensive work on this subject ever written. He was a successful and much appreciated practitioner of dentistry; a thorough and beloved teacher; one of the greatest students of the science and art of dentistry; a most versatile and capable contributor to dental literature; a constant attendant and contributor to the programmes of most of our important Dental Societies; and most of all a good friend to every honest and well intentioned worker in any line of dental research. At his age it was not possible that the profession should expect more from his great intellectual storehouse, and there is no question that Dr. Black will always be remembered for the great contributions he made to the profession, although future generations will not miss the inspiration of this great man so much as many who have known him in the class-room and in dental organizations all over the country. A great spirit has gone from us.

